# A novel and superior Lasso-plate technique in treatment for coronoid process fracture in the terrible triad of elbow

**DOI:** 10.1038/s41598-023-38885-1

**Published:** 2023-07-19

**Authors:** Chaofeng Wang, Congming Zhang, Dawei Zhou, Dongxing Lu, Zhong Li, Ning Duan, Kun Zhang

**Affiliations:** Department of Orthopaedic Surgery, Hong Hui Hospital, Xi’an Jiaotong University College of Medicine, NO. 76 Nanguo Road, Beilin District, Xi’an, 710054 Shaanxi Province China

**Keywords:** Diseases, Trauma

## Abstract

The treatment of ulna coronal process fractures in the terrible triad of elbow, especially type I and II Regan–Morrey coronoid fractures, still have been controversial. The purpose of this retrospective study was to evaluate the novel Lasso-plate technique to have a more reliable fixation and a well clinical outcomes for type I and II Regan–Morrey coronoid fractures in a terrible triad of the elbow (TTE). Patients with simple TTE, closed fracture, aged > 18 years, duration of injury < 2 weeks, type I and II Regan–Morrey coronoid process fracture fixed by the Lasso-plate technique or ORIF were enrolled in the study. Total 144 patients with type I and II Regan–Morrey coronoid fracture in TTE were included in the Lasso-plate group or ORIF (open reduction and internal fixation) group in the Xi’an Honghui Hospital from January 2017 to December 2020. Eighty-six patients in Lasso-plate group underwent surgery using a novel Lasso-plate technique. And other 58 patients in ORIF group underwent surgery using ORIF. The data of two groups, including the X-ray films, Computed tomography (CT), the range of elbow motion, Mayo Elbow Performance Score (MEPS) and the surgical complications, were extracted from the hospital’s patient records. All patients in both groups were followed up at least 12 months. The mean operation time (88.2 ± 12.3 min) in Lasso-plate group is shorter than that of ORIF group (109.1 ± 13.0 min). There was one patient with injury of deep branch of radial nerve and one patient with superficial surgical incision infection in Lasso-plate group. There were two patients with surgical incision infection in ORIF group. There were three heterotopic ossifications in Lasso-plate group and eight heterotopic ossifications in ORIF group. There were 5 elbow joints stiffness in Lasso-plate group and 12 in ORIF group. At 12 months follow up, the mean range of flexion–extension motion in Lasso-plate group was 122.9° ± 13.4° versus 113.2° ± 18.1° in ORIF group (p < 0.01), the mean 89.7 ± 5.6 MEPS in Lasso-plate group versus mean 83.7 ± 6.1 MEPSin ORIF group. The fixation of coronoid process fracture in TTE by the Lasso-plate technique, especially type I and II Regan–Morrey coronoid fracture, could be easier to master and operate, could provide the sufficient stability of elbow joint to enable early functional exercise, along with a better clinical outcome, a lower surgical complication. For the treatment of TTE, we recommend the fixation of type I and II Regan–Morrey coronoid fracture with the Lasso-plate technique, which would result in a better clinical outcome.

## Introduction

The terrible triad of elbow (TTE) was first described by Hotchkiss^1^ in 1996, which is defined as the fracture of the radial head and the coronal process of ulna combined with the dislocation of the elbow. The treatment of TTE is great challenge for orthopedic surgeons as often having the poor clinical outcomes, such as elbow stiffness, instability, pain, multiple reoperations and traumatic arthritis, etc.^2–4^.

The loss of bone support and the injury of soft tissues, such as the fracture of coronal process and radial head, collateral ligament injury and tear of anterior joint capsule would lead to the instability of elbow joint^5–7^. Although some scholars have proposed the principle of conservative treatment, most scholars thought that the restoration of stability of the elbow by surgical skill is the key to the treatment of TTE^8,9^. Some studies^10–12^ also showed that early functional rehabilitation training of elbow joint is another key to treatment of TTE. The repair or reconstruction of the radial head and repair of the LUCL are technically feasible. The fracture of radial head is often treated by open reduction and internal fixation (ORIF) or radial head arthroplasty. The injury of collateral ligament is often restored by anchor suture or a hinged external fixation. However, the treatment of ulna coronal process fracture still remains challenging. The fixation of the coronoid fracture has some options, such as suture lasso technique^9,13^, lag screws^14^, microplates^15^, suture anchors^13,16^. For big fracture fragments, the microplate or lag screw could provide the stable fixation and have a good outcome. However, for small fracture fragments or comminuted fractures, microplates and screws could not effectively and reliably fix, even lead to splitting of bone fragment. The suture lasso technique and suture anchors, although effective, have the potential to cause lacerations and result in unstable biomechanical fixation. In order to overcome the disadvantages, we used a novel Lasso-plate technique to fix type I and II Regan–Morrey coronoid process fracture in the terrible triad of the elbow from 2011 in the Xi’an Honghui Hospital.

The purpose of our study was to evaluate the novel Lasso-plate technique to have or not a more reliable fixation and a well clinical outcomes for type I and II Regan–Morrey coronoid fracture in a terrible triad of the elbow. We hypothesized that the novel Lasso-plate technique would be superior to ORIF with respect to clinical outcome and postoperative complications.

## Materials and methods

After ethics committee approval, a retrospective review of institutional databases was performed in Xi’an Honghui Hospital. The data of all patients who had been treated for TTE in the Xi’an Honghui Hospital between January 2017 to December 2020 were collected. All patients were treated according to a standard surgical algorithm that was consistent with previously published protocols^8,14,17,18^. We confirmed that all methods were carried out in accordance with relevant guidelines and regulations. We confirm that all study protocols were approved by the Xi’an Honghui Hospital Research Ethics Committee.

Patients with simple TTE, closed fracture, aged > 18 years, duration of injury < 2 weeks, type I and II Regan–Morrey coronoid process fracture fixed by the Lasso-plate technique or ORIF were enrolled in the study, whereas patients with compound fracture, vascular or nerve injuries, poly-trauma, history of elbow surgery or for less than 12 months follow-up were excluded from the study. In 2017–2018, we utilized the ORIF technique for treating patients with TTE coronary process fractures. However, in 2019–2020, after inventing the Lasso-plate technique, nearly all patients with TTE coronary process fractures were treated using the Lasso-plate technique. Eighty-six patients with coronoid process fracture fixed by the Lasso-plate technique were included in Lasso-plate group. Fifty-eight patients with coronoid process fracture fixed by ORIF were included in ORIF group. Total 144 patients were enrolled in the study.

### Surgical technique

Under general anesthesia combined with brachial plexus block anesthesia, the patient was placed in the supine position on operation table, and the affected arm is abducted and placed on the fluoroscopic operating table. The tourniquet was used. The lateral and dorsal elbow approaches were used routinely. The elbow was placed in the flexion supination position. The radial head and coronal process were exposed through lateral extensor digitorum communis (EDC) split approach, which starting from the anterolateral condyle of the lateral humerus, and cut along the center of the EDC. The elbow was flexed to easily expose the coronoid process and articular surface of the trochlea. The stability of elbow was restored by sequentially starting with the coronoid process, followed by the radial head and finally the lateral collateral ligament (LCL).

**Figure 1:**
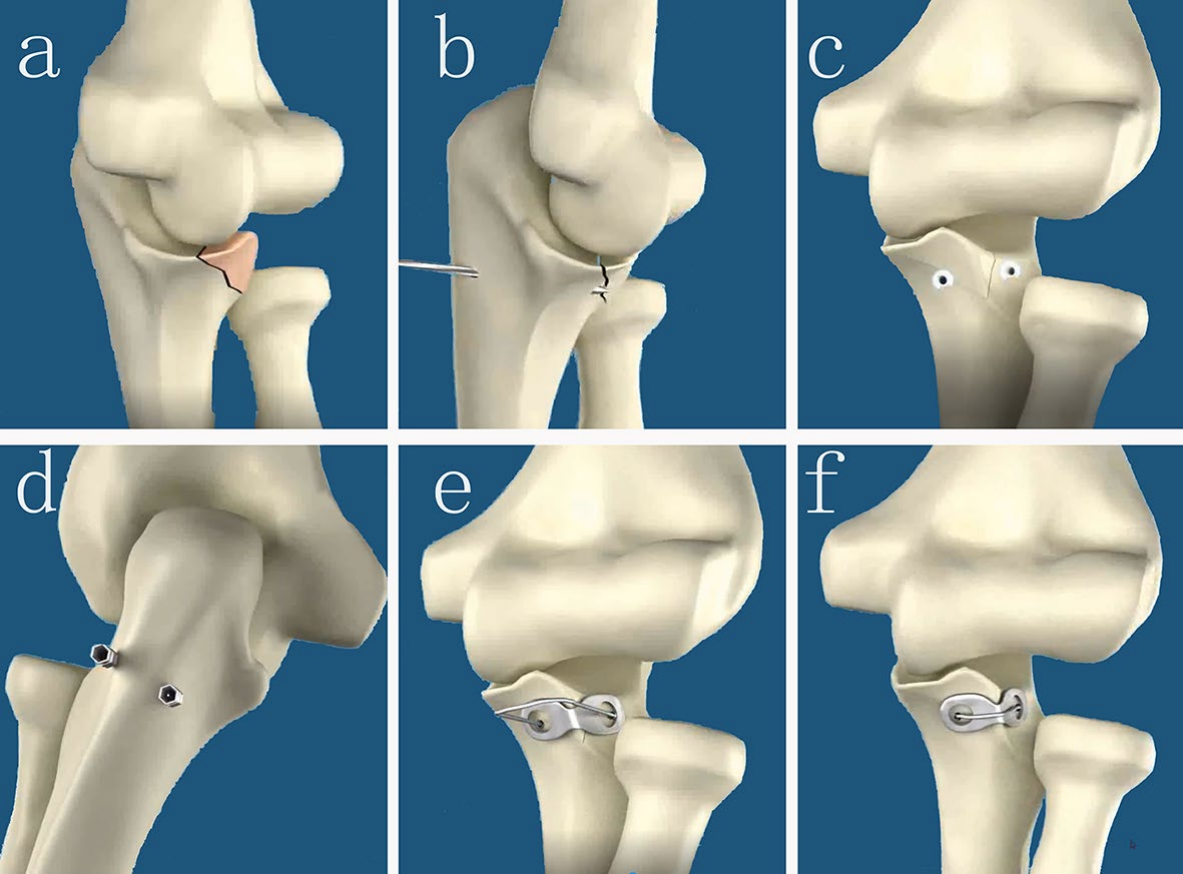
Schematic of Lasso-plate surgical technique. (**a**) Type II Regan–Morrey coronoid fractures. (**b**) A 2.0-mm K-wire or drill was used to drill the tunnels from dorsal ulna to coronoid process. (**c**) The tunnels were located on both sides of the fracture fragment of coronal process fracture. (**d**) Then to take a hollow lumbar puncture needle and insert it into the bone tunnel. (**e**) The Lasso-plate was placed in the gap between the coronoid and the anterior capsule, then the wire tails of Lasso-plate were introduced to passing through the tunnels from the anterior to the posterior. (**f**) The fracture was reduced and fixed when the steel wire was tightened.

Coronal process fracture was fixed by Lasso-plate technique or ORIF. For Lasso-plate technique (Fig. 1), coronal process fracture was reduced through the lateral incision. First a longitudinal incision was made along the ulnar dorsal ridge, about 1–2 cm in length. A 2.0-mm K-wire or drill was used to drill the tunnels from the points of 0.5 cm to the ulnar crest bilaterally to the points of both sides of the coronoid process. When the fragment of fracture is big, the tunnel could pass through fragment of coronal process fracture. When the fragment of fracture is small or comminuted, the tunnels should be located on both sides of the fracture fragment of coronal process fracture to avoid the iatrogenic fracture. The Lasso-plate was made with a 1.0-mm stainless steel wire placed through the two ends holes of 2 or 3 mini-plate, which were plasticized according to the anatomical shape of coronal process. Then to take a hollow lumbar puncture needle and insert it into the bone tunnel, the Lasso-plate was placed in the gap between the coronoid and the anterior capsule, and the wire tails of Lasso-plate were introduced to passing through the tunnels from the anterior to the posterior. Finally, the steel wire was tightened to reduce and fix the fracture. For the ORIF technique, coronal process fracture was fixed routinely by lag screws or mini-plates.

Radial head fracture was treated usually by fixation of lag screw or miniplate. When the radial head fracture was too comminated to be fixed, radial head replacement was used to treat. LCL was repaired or reconstructed with suture anchors or bone drilling sutures.

After fixation of the coronoid, fixation or replacement of radial head and repair of the LCL complex was accomplished, the stability of the elbow was evaluated by using the hanging arm test^13,15,19^. From the perspective of intraoperative lateral view, if concentric reduction of humeral ulnar joint could be maintained with the weight of the hand and forearm acting as a dislocating force, the elbow was thought to be the stability. When instability still existed, the medial collateral ligament (MCL) was repaired by the suture anchor through a medial approach, or the hinged external fixators were placed to maintain the concentric joint reduction. The hinged fixators were displaced after six weeks. The wound was placed a perfusion irrigation drainage tube and closed in layers.

### Postoperative protocol

Intravenous infusion of cefuroxime sodium was lasted for 24 h postoperatively to prevent the surgical site infection (SSI). After the surgery, all patients were prescribed 25 mg of indomethacin thrice daily for six weeks to prevent heterotopic ossification (HO). After the operation, the elbow joint was immobilized by a brace with 90° of flexion for three days. The irrigation drainage tube was removed 2 ~ 4 days after operation. As pain and swelling subsided from the 4th days after surgery, exercise motion of elbow was done and taught by the physical therapist. The range of elbow motion was limited to 30°–120° in the first six weeks. Then the range of the motion was gradually increased from the 7th weeks.

### Data measurement

X-ray film, CT were performed 3 days after operation to evaluate the effect of reduction and fixation. The X-ray films of elbow joint were examined at 4, 12, 24 weeks and 1 year after operation for joint congruency, heterotopic ossification, union time, and implant. Fracture healing was defined as the disappearance of the fracture line or the trabecular bone passing through the fracture line in X-ray film. Fracture healing in X-ray film were determined by two senior orthopedic doctors. During follow-up, the range of motion (ROM) of the elbow was recorded, including elbow flexion, extension, and forearm pronation and supination. “The stiffness of elbow joint was defined as flexion–extension or rotation range of motion < 100°^20^.” At the 1 year follow-up, elbow function was evaluated by Mayo Elbow Performance Score (MEPS)^21,22^. The data of two groups, including the X-ray films, CT, the range of elbow motion, MEPS and the surgical complications, were extracted from the hospital’s patient records.

### Statistical methods

The statistical software package SPSS 26.0 was used to analyze the results. Descriptive statistics were provided for all baseline characteristics and study endpoints. Quantitative variables were documented as the mean ± standard deviation. Before statistical analysis, all quantitative variables were tested for Normal distribution. Quantitative variables conforming to normal distribution in the two groups were assessed by independent Student's t test, while qualitative data between two groups were assessed by either the chi-square test or Fisher’s exact test. A p value < 0.05 was considered statistically significant.

### Ethical approval

The Xi’an Honghui Hospital Research Ethics Committee has confirmed this study to conform ethical principle (No.202202018).

### Consent to participate

Informed consent was obtained from all individual participants included in the study.

### Consent to publish

**Figure 2:**
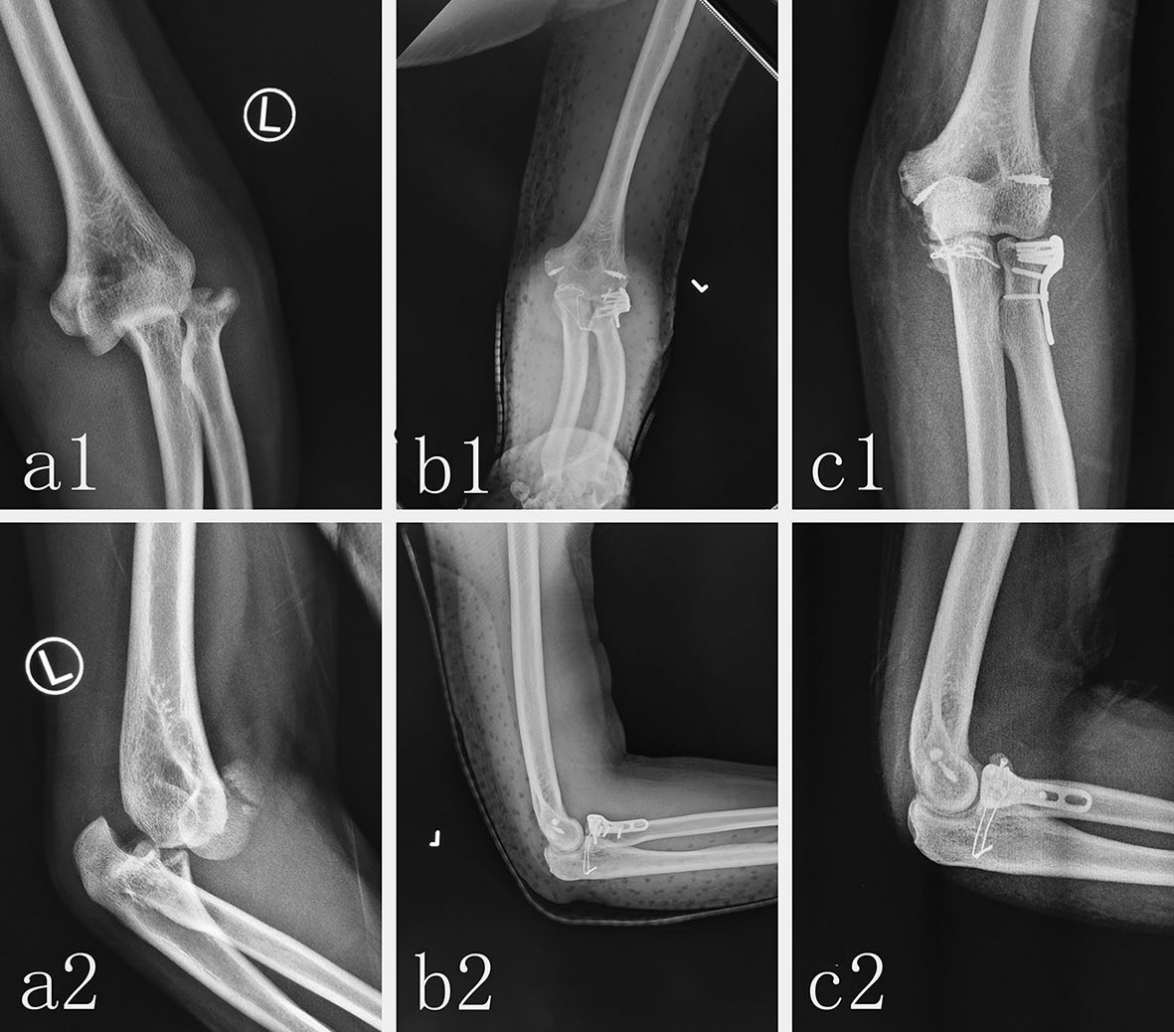
Radiological image of typical patient with TTE using Lasso-plate technique. The preoperative anteroposterior X-ray (**a1**) and lateral X-ray (**a2**) showed the posterior dislocation of left elbow joint, the fracture of radial head and coronoid process. An anteroposterior X-ray (**b1**) and lateral X-ray (**b2**) at postoperative follow-up showed that the fracture of coronoid process was reduced and fixed by Lasso-plate technique, radial head fracture was fixed by miniplate, and the LCL of elbow were repaired by suture anchor. An anteroposterior X-ray (**c1**) and lateral X-ray (**c2**) at 6-months postoperative follow-up showed that fracture had healed well and the dislocation and instability of elbow were not observed.

**Figure 3:**
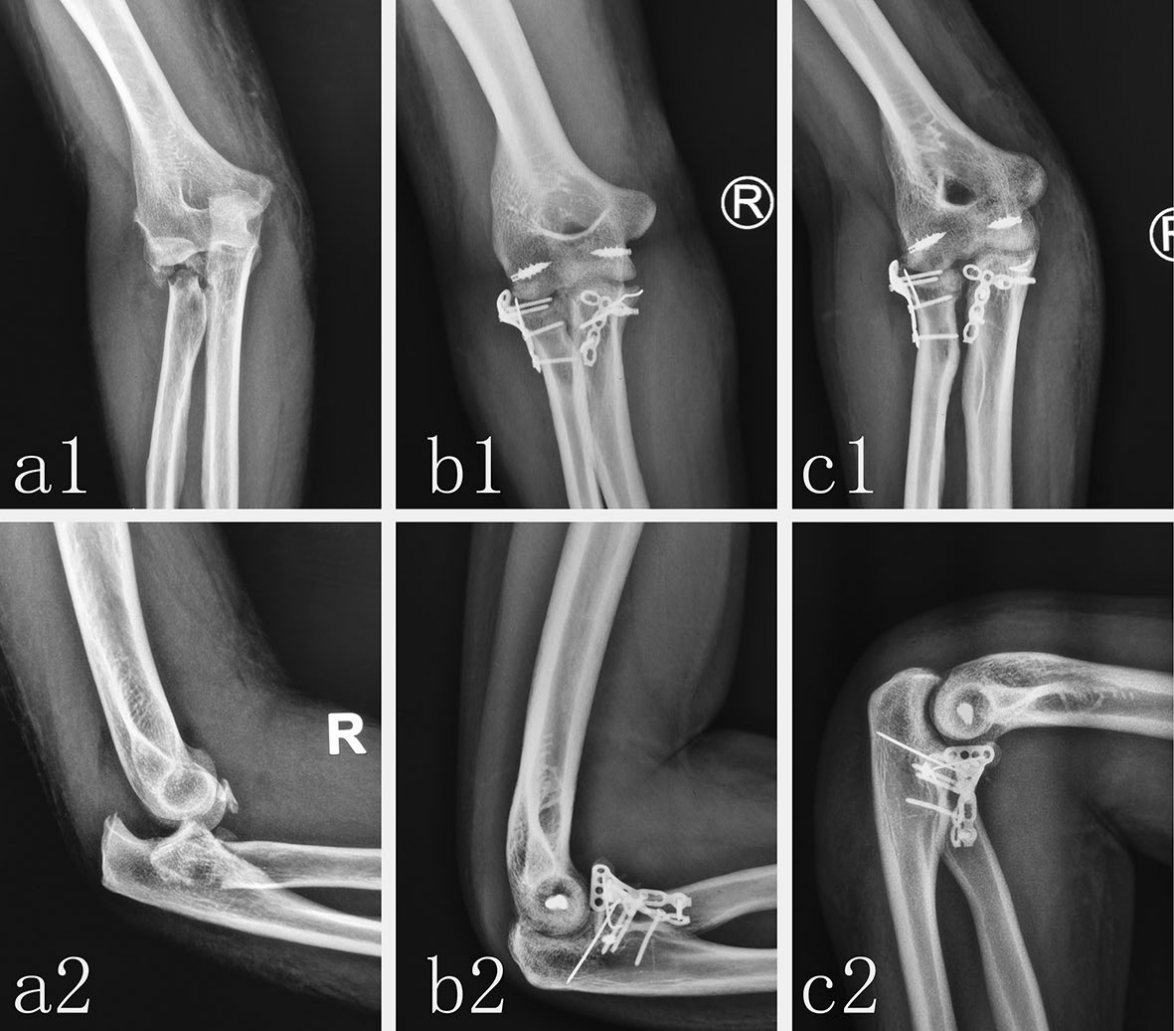
Radiological image of typical patient with TTE using ORIF. The preoperative anteroposterior X-ray (**a1**) and lateral X-ray (**a2**) showed the posterior dislocation of right elbow joint, the fracture of radial head and coronoid process. An anteroposterior X-ray (**b1**) and lateral X-ray (**b2**) at postoperative follow-up showed that the fracture of coronoid process and radial head were fixed by miniplate and the LCL and MCL of elbow were repaired by suture anchor. An anteroposterior X-ray (**c1**) and lateral X-ray (**c2**) at 6-months postoperative follow-up showed that fracture had healed well and the dislocation and instability of elbow were not observed.

The authors affirm that human research participants provided informed consent for publication of the images in Figs. 2 and 3.

## Results

The data of total 144 patients were collected. Main characteristics of the patients in both groups are no significantly difference (Table 1). All patients in both groups were followed up at least 12 months (mean 15 months, range from 12 to 38 months). The mean operation time (88.2 ± 12.3 min) in Lasso-plate group is shorter than that of ORIF group (109.1 ± 13.0 min) (Table 1). One patient in Lasso-plate group had the injury of deep branch of radial nerve. In the absence of surgical intervention, the radial nerve function completely recovered after 2 months. No patient in ORIF group had the main nerve or vascular injury. Three patients (1 in Lasso-plate technique group and 2 in ORIF group) had the superficial surgical incision infection. The rate of incision infection showed no significant difference between the two groups. All three patients were cured by dressing change or debridement.

**Table 1:** Main characteristics of the patients and operation time in Lasso-plate and ORIF groups.

Characteristics	Lasso-plate (86)	ORIF (58)	p value^**#**^
Age	44.4 ± 15.9	48.2 ± 17.2	0.239
Sex (male)	93	72	0.370
Operation time (min)	88.2 ± 12.3	109.1 ± 13.0	< 0.001

^**#**^A p value < 0.05 was considered statistically significant.

There were three heterotopic ossifications of elbow joint that occurred in the eighty-six elbows in Lasso-plate group by X-ray, which was significantly less than eight heterotopic ossifications of elbow joint that occurred in the fifteen-eight elbows in ORIF group (p = 0.022). Fracture healing was defined as the disappearance of the fracture line or the trabecular bone passing through the fracture line in X-ray film. Fracture healing in X-ray film were determined by two senior orthopedic doctors. The mean healing time in Lasso-plate group (mean 14.7 ± 1.9 weeks) showed no significant difference compared to that (mean 14.1 ± 1.7 weeks) in ORIF group. The X-ray images of the typical patient treated in Lasso-plate group have been shown in Fig. 2. The X-ray images of another typical patient treated in ORIF group have been shown in Fig. 3.

The stiffness of elbow joint was defined as flexion–extension or rotation range of motion < 100°^22^. During the follow up, there were five elbow joints with stiffness that occurred in the eighty-six elbows in Lasso-plate group, which was significantly less than twelve elbow joints with stiffness that occurred in the fifty-eight elbows in ORIF group (p = 0.007). Twelve of fifteen patients suffered elbow joint stiffness were given to the release of elbow joint and have a satisfactory motion range. Another two patients tolerated the unsatisfactory elbow function. The data of main complications in Lasso-plate and ORIF groups were showed in Table 2.

**Table 2:** Comparison of the clinical outcomes and main complications between Lasso-plate and ORIF groups.

Clinical outcomes and main complications	Lasso-plate (86)	ORIF (58)	p value^**#**^
Heterotopic ossification	3	8	0.022
Elbow stiffness	5	12	0.007
Nerve or vascular injury	1	0	0.410
Superficial SSI*	1	2	0.346

^**#**^A p value < 0.05 was considered statistically significant.

*SSI Surgical Site Infection.

At 12 months follow up, the mean range of flexion–extension motion in Lasso-plate group was 122.9° ± 13.4°, which is significantly larger than 113.2° ± 18.1° in ORIF group (p < 0.001). The mean extension 11.1° ± 3.4° in Lasso-plate group was greater than 22° ± 4.7° in ORIF group. However, the mean flexion 134.0° ± 12.4° in Lasso-plate group is no significant difference to 135.2° ± 14.6° in ORIF group. But the mean range of rotation motion in both groups is no significant difference (147.5 ± 8.0 in Lasso-plate group and 145.5 ± 8.7 in ORIF respectively). At 12 months follow up, the mean MEPS was significantly higher than that of ORIF group (83.7 ± 6.1 > 89.7 ± 5.6, p < 0.001). The data of clinical outcomes in Lasso-plate and ORIF groups were shown in Table 3.

**Table 3:** Comparison of the clinical outcomes between Lasso-plate and ORIF groups.

Clinical outcomes and main complications	Lasso-plate (86)	ORIF (58)	p value^**#**^
Healing time (weeks)	14.7 ± 1.9	14.1 ± 1.7	0.082
Flexion–extension motion	122.9 ± 13.4	113.2 ± 18.1	< 0.001
MEPS* scores	89.7 ± 5.6	83.7 ± 6.1	< 0.001
Rotation motion	147.5 ± 8.0	145.5 ± 8.7	0.165

^#^A p value < 0.05 was considered statistically significant.

*MEPS Mayo Elbow performance Score.

## Discussion

Since TTE was first described by Hotchkiss in 1996, some studies and methods were reported to treat TTE and also made great achievements. However, the treatment of TTE is still great challenge for orthopedic surgeons. A standard surgical protocol to TTE play a very important role to improve the clinical outcome of patient suffered TTE^8,14,17,18,23^. Almost all surgeries have a consensus to the repair of the radial head fracture and LCL. The fracture of radial head is often treated by open reduction and internal fixation (ORIF) or radial head arthroplasty. The injury of collateral ligament is often restored by anchor suture or a hinged external fixation. However, the treatment of ulna coronal process fracture, especially type I and II Regan–Morrey coronoid fracture, still have been controversial.

Some studies^2,24^ suggested that the coronoid process have a very important role in the instability of a terrible triad injury of elbow. Schneeberger’s study^25^ reported that when the coronal process fractures, the humeral trochlear has the tendency of anterior medial dislocation due to the loss of anterior bone block, which affects the stability of the elbow joint. When the height of coronal process fracture is 30%, it will affect the stability of elbow joint, and when it is > 50%, it will obviously lead to instability of elbow joint. The anterior capsule of elbow joint is attached to the distal end of the coronal process at 6.5 mm, while tendon of biceps brachii is attached to the distal end of the coronal process at 11 mm. Therefore, when the fracture height of the coronal process is > 30%, they thought that it is necessary to repair the anterior bone and soft tissue stability. Most of the studies recommended the reconstruction of both radial head and coronoid process fractures in terrible triad injuries^18,23,26,27^. However, some clinical studies^28,29^ have reported that at short-term and mid-term follow up, type I or II coronoid process fracture according to Regan–Morrey classification can be stable if the radial head and ligamentous complexes are completely restored. In Antoni’s experience^30^, re-attaching the anterior capsule or the coronoid process fracture in TTE did not improve the clinical or radiographic outcomes at more than 4 years follow-up. To our clinical experience, we think that the fixation of coronoid process fracture is very important to provide an early stability of elbow joint and enable the early functional exercise. Our study results also confirmed that the fixation of coronoid process fracture resulted in a good clinical outcome, regardless of Lasso-plate technique or ORIF.

The fixation of ulna coronal process fracture has some methods including lag screw fixation, micro plate fixation, anchor screw fixation and suture lasso technique. For the most common small coronal process tip fracture or tip comminuted fracture, the use of screw fixation is easy to cause the iatrogenic fracture^13^, which is difficult to be accurately and effectively fixed. Garrigues and his colleagues^13^ reported that greater stability with fewer complications was achieved with use of the suture lasso technique for coronoid fracture fixation for TTE. However, in our clinical practice, we found the suture lasso technique have a lower stability of elbow and a poorer ability to prevent the displacement of fracture. A cadaveric biomechanical study also show that the suture lasso technique may provide less resistance to displacement of the coronoid compared with a lag screw technique. This result is consistent with our clinical findings. Therefore, we used the novel Lasso-plate technique to fix the coronoid process fracture. Compared with suture lasso technique, Lasso-plates plays a role as a buttress plate^31^. The Lasso-plate increases the contact area between the soft tissue and implant and reduces the pressure between the wire and capsule insertion^32^. The Lasso-plate technique provided an excellent biomechanical environment to the stability of elbow joint, which would enable early functional exercise. Our study results show that at 12 months follow up, the mean range of flexion–extension motion in Lasso-plate group was 126° versus 112° in ORIF group, the mean extension 11° in Lasso-plate group versus 22° in ORIF group, and the mean MEPS in Lasso-plate group was 88.6 versus mean 82.2 in ORIF group. The higher MEPS score and larger range of elbow motion arc in Lasso-plate technique group suggested the better the clinical outcome than that in ORIF group. We thought that in Lasso-plate technique group enabling early functional exercise is one main reason that the clinical outcomes of is better to that of ORIF.

In our study, the mean operation time in Lasso-plate group is significantly shorter than that of ORIF group (98.4 min < 116.7 min, p < 0.001), which suggested that the fixation of coronoid process fracture by Lasso-plate technique is easier than the fixation by lag screw or mini-plate. Easy to master and operate technology can reduce iatrogenic damage. The shorter operation time and the less operation damage were thought to be another main reason for the better clinical outcomes in Lasso-plate technique group.

There were some limitations to our study. One limitation is that the non-randomized controlled grouping and the involvement of different surgeons may have had a partial impact on the research results. Another limitation is the use of various implants for coronoid process fracture, radial head fracture, and LCL reconstruction, which could lead to slight bias. These limitations could be addressed by designing a prospective randomized controlled study for future research.

## Conclusion

TTE is a serious injury in clinic and a challenging fracture-dislocation of the elbow to treat. The fixation of coronoid process fracture by the Lasso-plate technique, especially type I and II Regan–Morrey coronoid fracture, could be easier to master and operate, could provide the sufficient stability of elbow joint to enable early functional exercise, along with a better clinical outcome, a lower surgical complication. For the treatment of TTE, we recommend the fixation of type I and II Regan–Morrey coronoid fracture with the Lasso-plate technique, which would result in a better clinical outcome.

## Data Availability

The datasets generated during and/or analysed during the current study are available from the corresponding author on reasonable request.
